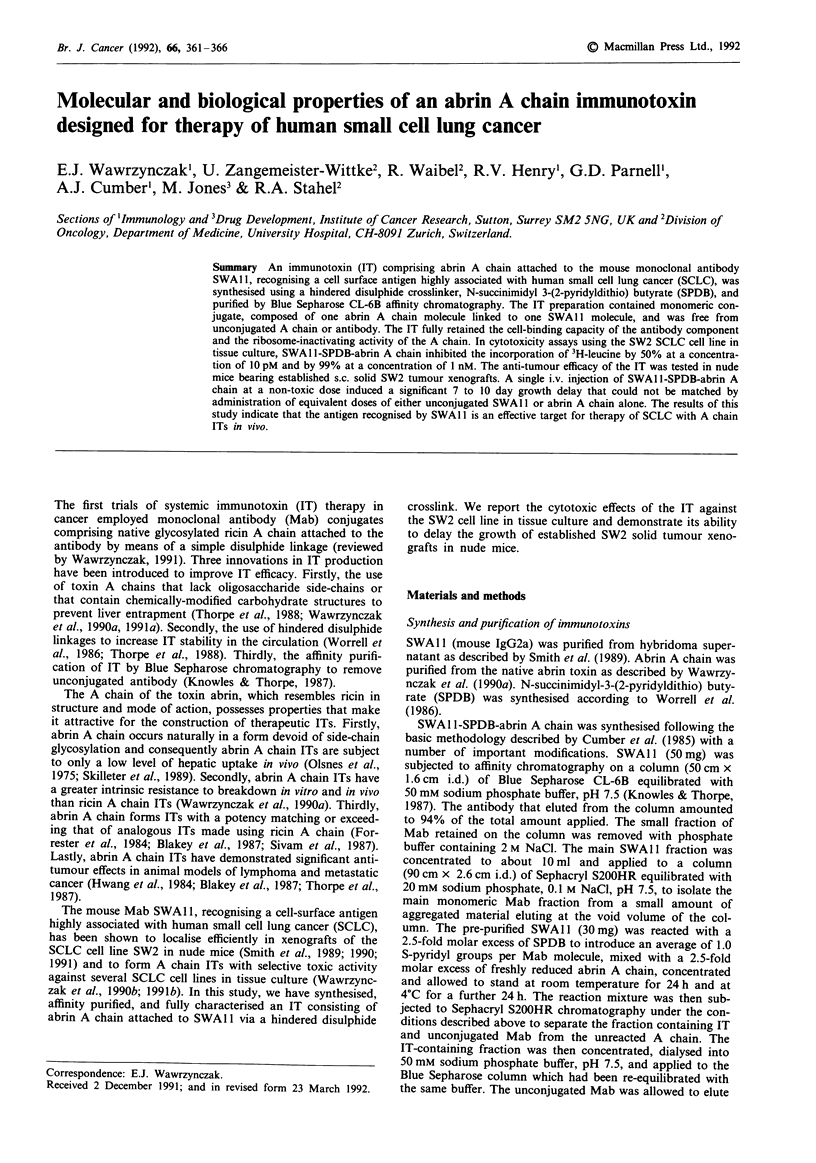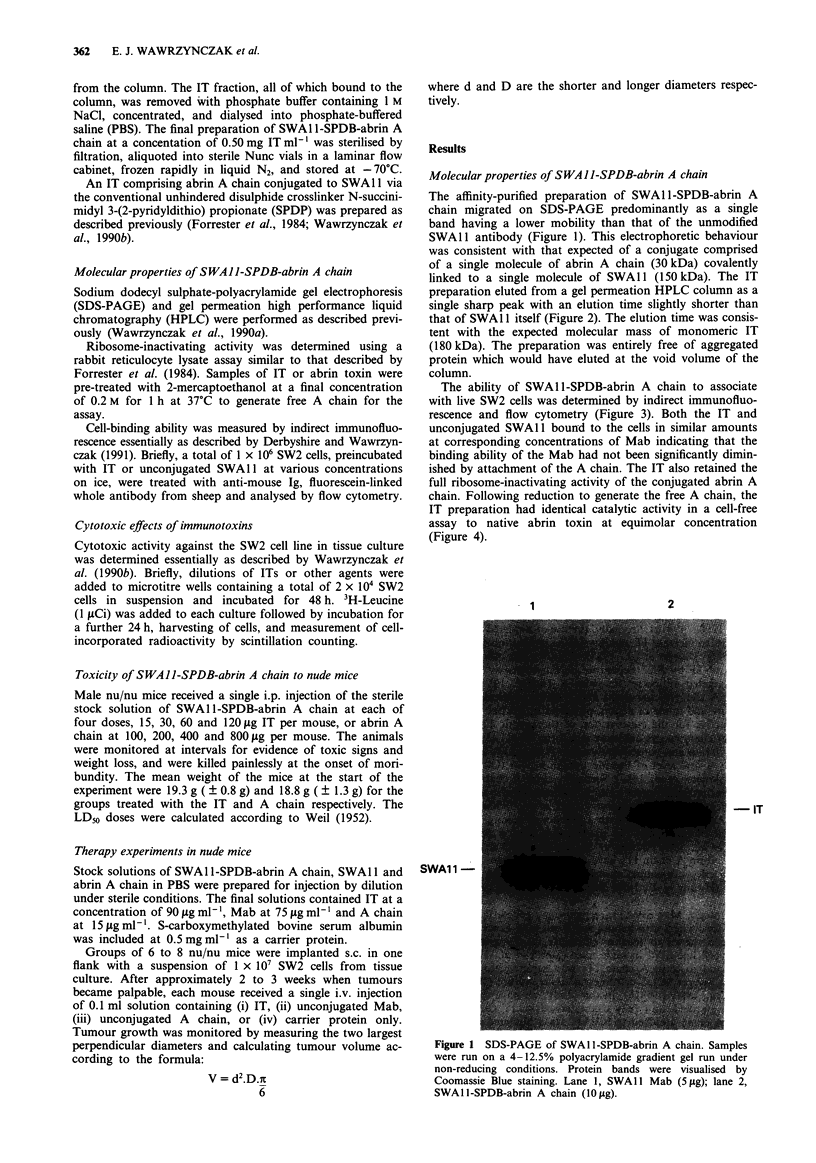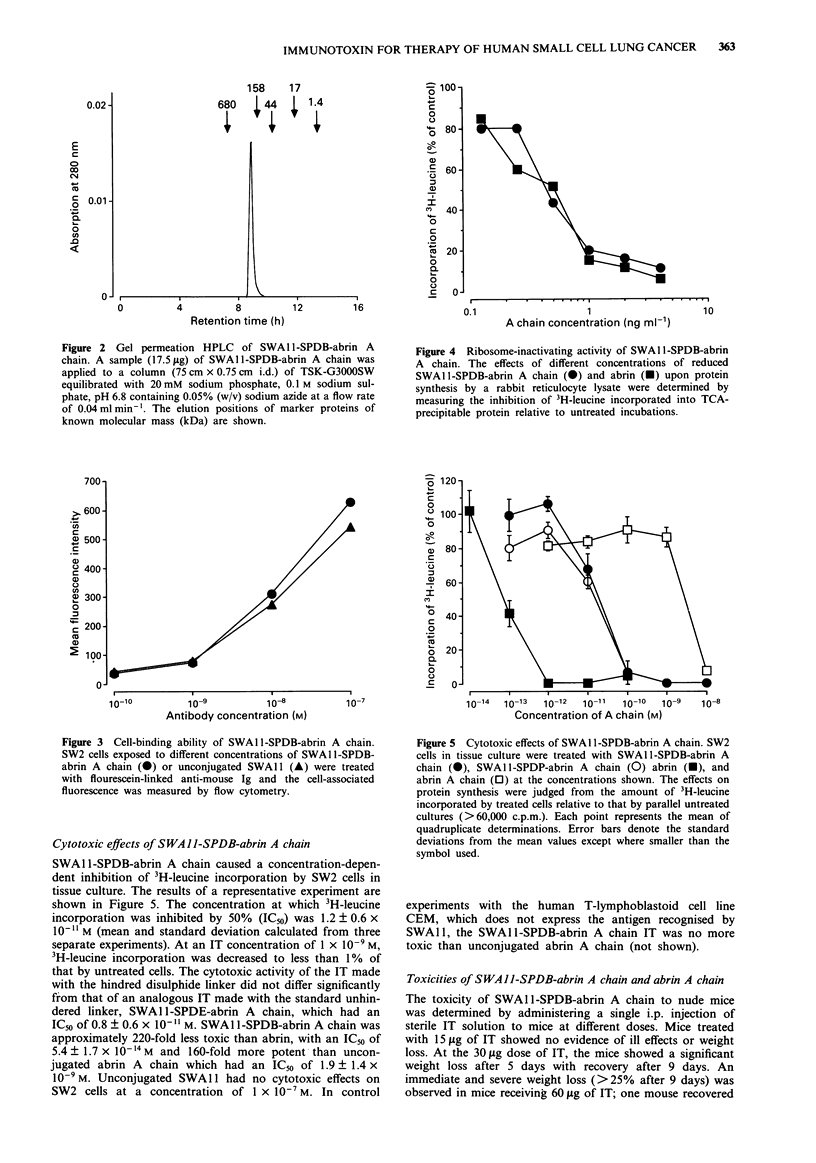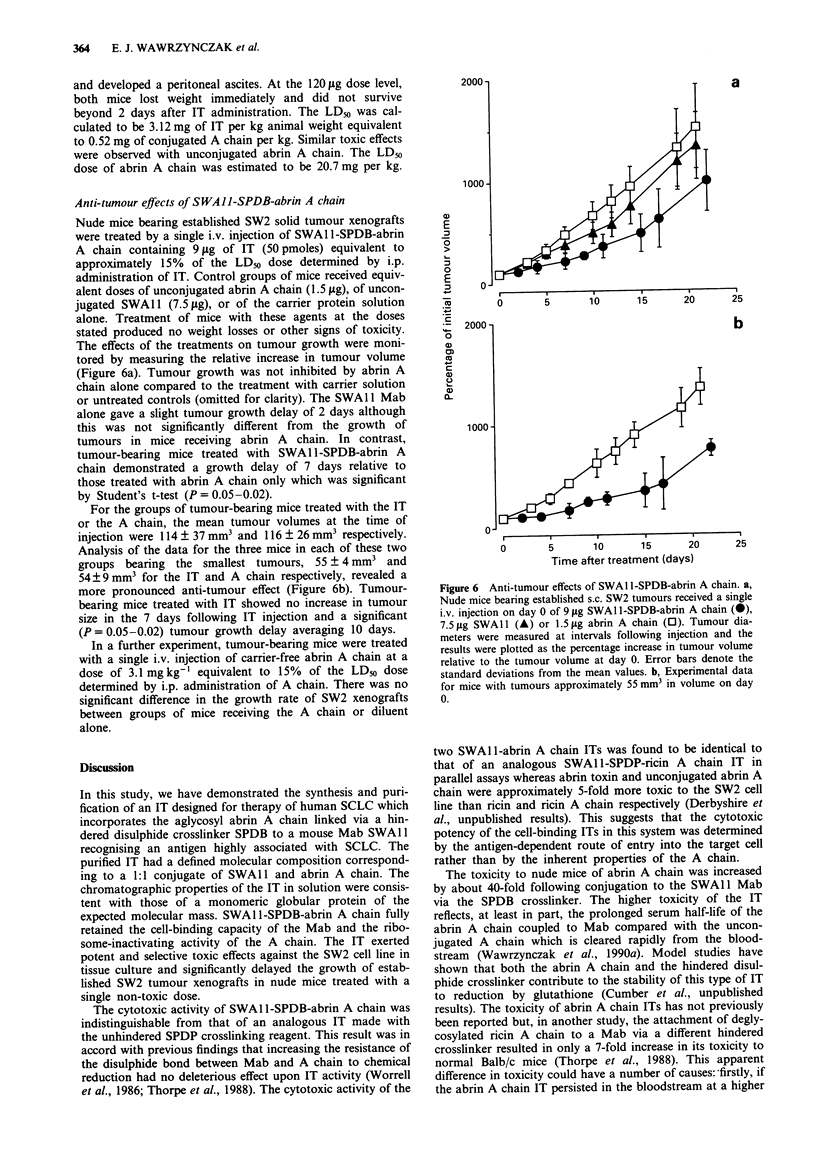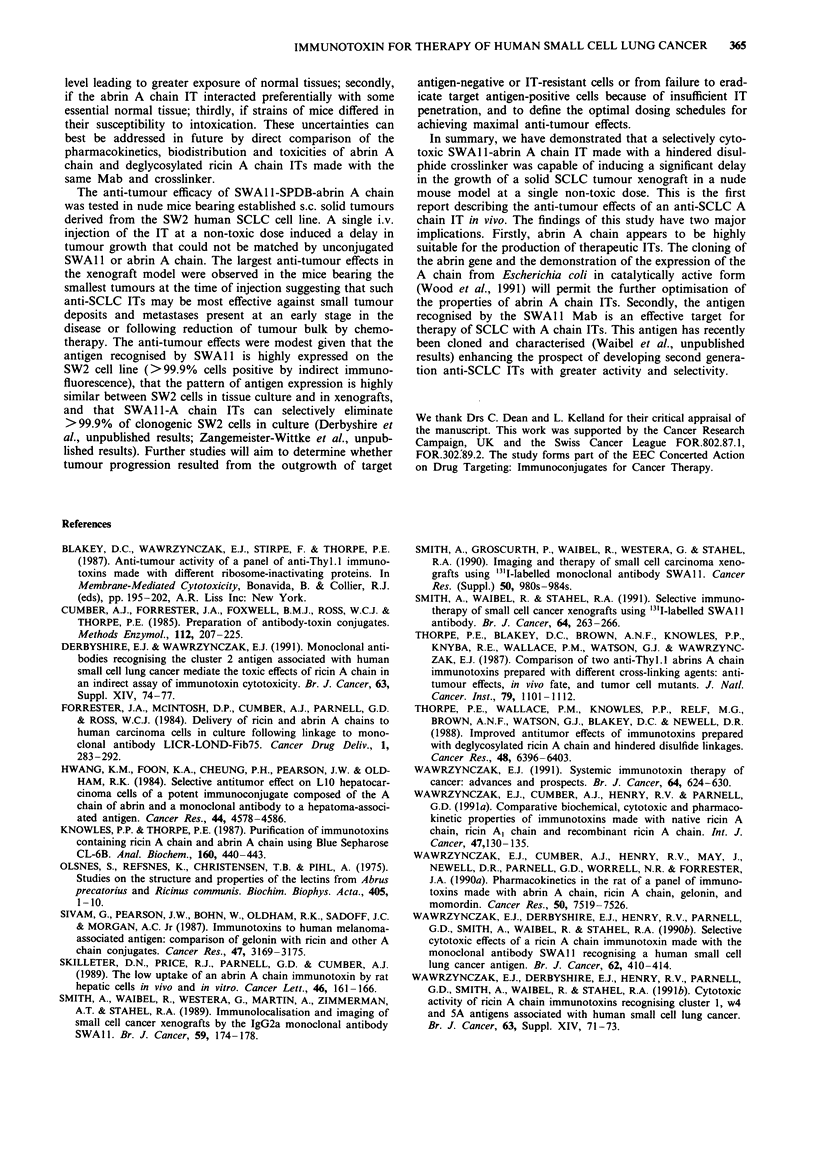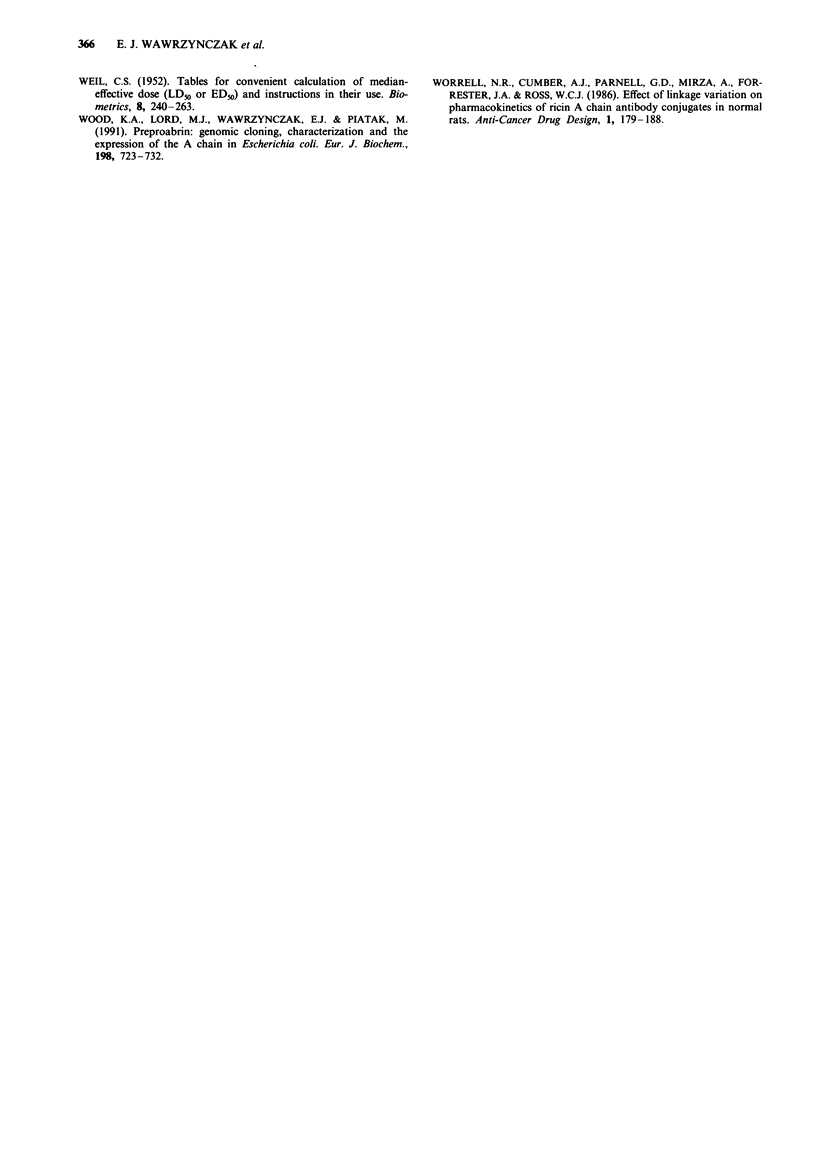# Molecular and biological properties of an abrin A chain immunotoxin designed for therapy of human small cell lung cancer.

**DOI:** 10.1038/bjc.1992.271

**Published:** 1992-08

**Authors:** E. J. Wawrzynczak, U. Zangemeister-Wittke, R. Waibel, R. V. Henry, G. D. Parnell, A. J. Cumber, M. Jones, R. A. Stahel

**Affiliations:** Section of Immunology, Institute of Cancer Research, Sutton, Surrey, UK.

## Abstract

**Images:**


*Br. J. Cancer* (1992), **66**, 361–366 © Macmillan Press Ltd., 1992

### Molecular and biological properties of an abrin A chain immunotoxin designed for therapy of human small cell lung cancer

E.J. Wawrzynczak[1], U. Zangemeister-Wittke[2], R. Waibel[2], R.V. Henry[1], G.D. Parnell[1], A.J. Cumber[1], M. Jones[3] & R.A. Stahel[2]

*Sections of [1]Immunology and [3]Drug Development, Institute of Cancer Research, Sutton, Surrey SM2 5NG, UK and [2]Division of Oncology, Department of Medicine, University Hospital, CH-8091 Zurich, Switzerland.*

**Summary** An immunotoxin (IT) comprising abrin A chain attached to the mouse monoclonal antibody SWA11, recognising a cell surface antigen highly associated with human small cell lung cancer (SCLC), was synthesised using a hindered disulphide crosslinker, N-succinimidyl 3-(2-pyridyldithio) butyrate (SPDB), and purified by Blue Sepharose CL-6B affinity chromatography. The IT preparation contained monomeric conjugate, composed of one abrin A chain molecule linked to one SWA11 molecule, and was free from unconjugated A chain or antibody. The IT fully retained the cell-binding capacity of the antibody component and the ribosome-inactivating activity of the A chain. In cytotoxicity assays using the SW2 SCLC cell line in tissue culture, SWA11-SPDB-abrin A chain inhibited the incorporation of $^{3}$H-leucine by 50% at a concentration of 10 pM and by 99% at a concentration of 1 nM. The anti-tumour efficacy of the IT was tested in nude mice bearing established s.c. solid SW2 tumour xenografts. A single i.v. injection of SWA11-SPDB-abrin A chain at a non-toxic dose induced a significant 7 to 10 day growth delay that could not be matched by administration of equivalent doses of either unconjugated SWA11 or abrin A chain alone. The results of this study indicate that the antigen recognised by SWA11 is an effective target for therapy of SCLC with A chain ITs *in vivo*.

The first trials of systemic immunotoxin (IT) therapy in cancer employed monoclonal antibody (Mab) conjugates comprising native glycosylated ricin A chain attached to the antibody by means of a simple disulphide linkage (reviewed by Wawrzynczak, 1991). Three innovations in IT production have been introduced to improve IT efficacy. Firstly, the use of toxin A chains that lack oligosaccharide side-chains or that contain chemically-modified carbohydrate structures to prevent liver entrapment (Thorpe *et al.*, 1988; Wawrzynczak *et al.*, 1990*a*, 1991*a*). Secondly, the use of hindered disulphide linkages to increase IT stability in the circulation (Worrell *et al.*, 1986; Thorpe *et al.*, 1988). Thirdly, the affinity purification of IT by Blue Sepharose chromatography to remove unconjugated antibody (Knowles & Thorpe, 1987).

The A chain of the toxin abrin, which resembles ricin in structure and mode of action, possesses properties that make it attractive for the construction of therapeutic ITs. Firstly, abrin A chain occurs naturally in a form devoid of side-chain glycosylation and consequently abrin A chain ITs are subject to only a low level of hepatic uptake *in vivo* (Olsnes *et al.*, 1975; Skilleter *et al.*, 1989). Secondly, abrin A chain ITs have a greater intrinsic resistance to breakdown *in vitro* and *in vivo* than ricin A chain ITs (Wawrzynczak *et al.*, 1990*a*). Thirdly, abrin A chain forms ITs with a potency matching or exceeding that of analogous ITs made using ricin A chain (Forrester *et al.*, 1984; Blakey *et al.*, 1987; Sivam *et al.*, 1987). Lastly, abrin A chain ITs have demonstrated significant anti-tumour effects in animal models of lymphoma and metastatic cancer (Hwang *et al.*, 1984; Blakey *et al.*, 1987; Thorpe *et al.*, 1987).

The mouse Mab SWA11, recognising a cell-surface antigen highly associated with human small cell lung cancer (SCLC), has been shown to localise efficiently in xenografts of the SCLC cell line SW2 in nude mice (Smith *et al.*, 1989; 1990; 1991) and to form A chain ITs with selective toxic activity against several SCLC cell lines in tissue culture (Wawrzynczak *et al.*, 1990*b*; 1991*b*). In this study, we have synthesised, affinity purified, and fully characterised an IT consisting of abrin A chain attached to SWA11 via a hindered disulphide crosslink. We report the cytotoxic effects of the IT against the SW2 cell line in tissue culture and demonstrate its ability to delay the growth of established SW2 solid tumour xenografts in nude mice.

Correspondence: E.J. Wawrzynczak.
Received 2 December 1991; and in revised form 23 March 1992.

### Materials and methods

### *Synthesis and purification of immunotoxins*

SWA11 (mouse IgG2a) was purified from hybridoma supernatant as described by Smith *et al.* (1989). Abrin A chain was purified from the native abrin toxin as described by Wawrzynczak *et al.* (1990*a*). N-succinimidyl-3-(2-pyridyldithio) butyrate (SPDB) was synthesised according to Worrell *et al.* (1986).

SWA11-SPDB-abrin A chain was synthesised following the basic methodology described by Cumber *et al.* (1985) with a number of important modifications. SWA11 (50 mg) was subjected to affinity chromatography on a column (50 cm × 1.6 cm i.d.) of Blue Sepharose CL-6B equilibrated with 50 mM sodium phosphate buffer, pH 7.5 (Knowles & Thorpe, 1987). The antibody that eluted from the column amounted to 94% of the total amount applied. The small fraction of Mab retained on the column was removed with phosphate buffer containing 2 M NaCl. The main SWA11 fraction was concentrated to about 10 ml and applied to a column (90 cm × 2.6 cm i.d.) of Sephacryl S200HR equilibrated with 20 mM sodium phosphate, 0.1 M NaCl, pH 7.5, to isolate the main monomeric Mab fraction from a small amount of aggregated material eluting at the void volume of the column. The pre-purified SWA11 (30 mg) was reacted with a 2.5-fold molar excess of SPDB to introduce an average of 1.0 S-pyridyl groups per Mab molecule, mixed with a 2.5-fold molar excess of freshly reduced abrin A chain, concentrated and allowed to stand at room temperature for 24 h and at 4°C for a further 24 h. The reaction mixture was then subjected to Sephacryl S200HR chromatography under the conditions described above to separate the fraction containing IT and unconjugated Mab from the unreacted A chain. The IT-containing fraction was then concentrated, dialysed into 50 mM sodium phosphate buffer, pH 7.5, and applied to the Blue Sepharose column which had been re-equilibrated with the same buffer. The unconjugated Mab was allowed to elute from the column. The IT fraction, all of which bound to the column, was removed with phosphate buffer containing 1 M NaCl, concentrated, and dialysed into phosphate-buffered saline (PBS). The final preparation of SWA11-SPDB-abrin A chain at a concentation of 0.50 mg IT $ml^{-1}$ was sterilised by filtration, aliquoted into sterile Nunc vials in a laminar flow cabinet, frozen rapidly in liquid $N_2$, and stored at $-70°C$.

An IT comprising abrin A chain conjugated to SWA11 via the conventional unhindered disulphide crosslinker N-succinimidyl 3-(2-pyridyldithio) propionate (SPDP) was prepared as described previously (Forrester *et al.*, 1984; Wawrzynczak *et al.*, 1990*b*).

### *Molecular properties of SWA11-SPDB-abrin A chain*

Sodium dodecyl sulphate-polyacrylamide gel electrophoresis (SDS-PAGE) and gel permeation high performance liquid chromatography (HPLC) were performed as described previously (Wawrzynczak *et al.*, 1990*a*).

Ribosome-inactivating activity was determined using a rabbit reticulocyte lysate assay similar to that described by Forrester *et al.* (1984). Samples of IT or abrin toxin were pre-treated with 2-mercaptoethanol at a final concentration of 0.2 M for 1 h at 37°C to generate free A chain for the assay.

Cell-binding ability was measured by indirect immunofluorescence essentially as described by Derbyshire and Wawrzynczak (1991). Briefly, a total of $1 \times 10^6$ SW2 cells, preincubated with IT or unconjugated SWA11 at various concentrations on ice, were treated with anti-mouse Ig, fluorescein-linked whole antibody from sheep and analysed by flow cytometry.

### *Cytotoxic effects of immunotoxins*

Cytotoxic activity against the SW2 cell line in tissue culture was determined essentially as described by Wawrzynczak *et al.* (1990*b*). Briefly, dilutions of ITs or other agents were added to microtitre wells containing a total of $2 \times 10^4$ SW2 cells in suspension and incubated for 48 h. $^3$H-Leucine (1 $\mu$Ci) was added to each culture followed by incubation for a further 24 h, harvesting of cells, and measurement of cell-incorporated radioactivity by scintillation counting.

### *Toxicity of SWA11-SPDB-abrin A chain to nude mice*

Male nu/nu mice received a single i.p. injection of the sterile stock solution of SWA11-SPDB-abrin A chain at each of four doses, 15, 30, 60 and 120 $\mu$g IT per mouse, or abrin A chain at 100, 200, 400 and 800 $\mu$g per mouse. The animals were monitored at intervals for evidence of toxic signs and weight loss, and were killed painlessly at the onset of moribundity. The mean weight of the mice at the start of the experiment were 19.3 g ($\pm$ 0.8 g) and 18.8 g ($\pm$ 1.3 g) for the groups treated with the IT and A chain respectively. The $LD_{50}$ doses were calculated according to Weil (1952).

### *Therapy experiments in nude mice*

Stock solutions of SWA11-SPDB-abrin A chain, SWA11 and abrin A chain in PBS were prepared for injection by dilution under sterile conditions. The final solutions contained IT at a concentration of 90 $\mu$g $ml^{-1}$, Mab at 75 $\mu$g $ml^{-1}$ and A chain at 15 $\mu$g $ml^{-1}$. S-carboxymethylated bovine serum albumin was included at 0.5 mg $ml^{-1}$ as a carrier protein.

Groups of 6 to 8 nu/nu mice were implanted s.c. in one flank with a suspension of $1 \times 10^7$ SW2 cells from tissue culture. After approximately 2 to 3 weeks when tumours became palpable, each mouse received a single i.v. injection of 0.1 ml solution containing (i) IT, (ii) unconjugated Mab, (iii) unconjugated A chain, or (iv) carrier protein only. Tumour growth was monitored by measuring the two largest perpendicular diameters and calculating tumour volume according to the formula:

$$V = d^2.D.\frac{\pi}{6}$$

where d and D are the shorter and longer diameters respectively.

### Results

### *Molecular properties of SWA11-SPDB-abrin A chain*

The affinity-purified preparation of SWA11-SPDB-abrin A chain migrated on SDS-PAGE predominantly as a single band having a lower mobility than that of the unmodified SWA11 antibody (Figure 1). This electrophoretic behaviour was consistent with that expected of a conjugate comprised of a single molecule of abrin A chain (30 kDa) covalently linked to a single molecule of SWA11 (150 kDa). The IT preparation eluted from a gel permeation HPLC column as a single sharp peak with an elution time slightly shorter than that of SWA11 itself (Figure 2). The elution time was consistent with the expected molecular mass of monomeric IT (180 kDa). The preparation was entirely free of aggregated protein which would have eluted at the void volume of the column.

The ability of SWA11-SPDB-abrin A chain to associate with live SW2 cells was determined by indirect immunofluorescence and flow cytometry (Figure 3). Both the IT and unconjugated SWA11 bound to the cells in similar amounts at corresponding concentrations of Mab indicating that the binding ability of the Mab had not been significantly diminished by attachment of the A chain. The IT also retained the full ribosome-inactivating activity of the conjugated abrin A chain. Following reduction to generate the free A chain, the IT preparation had identical catalytic activity in a cell-free assay to native abrin toxin at equimolar concentration (Figure 4).

**Figure 1** SDS-PAGE of SWA11-SPDB-abrin A chain. Samples were run on a 4–12.5% polyacrylamide gradient gel run under non-reducing conditions. Protein bands were visualised by Coomassie Blue staining. Lane 1, SWA11 Mab (5 $\mu$g); lane 2, SWA11-SPDB-abrin A chain (10 $\mu$g).

**Figure 2** Gel permeation HPLC of SWA11-SPDB-abrin A chain. A sample (17.5 μg) of SWA11-SPDB-abrin A chain was applied to a column (75 cm × 0.75 cm i.d.) of TSK-G3000SW equilibrated with 20 mM sodium phosphate, 0.1 M sodium sulphate, pH 6.8 containing 0.05% (w/v) sodium azide at a flow rate of 0.04 ml $min^{-1}$. The elution positions of marker proteins of known molecular mass (kDa) are shown.

**Figure 4** Ribosome-inactivating activity of SWA11-SPDB-abrin A chain. The effects of different concentrations of reduced SWA11-SPDB-abrin A chain (●) and abrin (■) upon protein synthesis by a rabbit reticulocyte lysate were determined by measuring the inhibition of $^3$H-leucine incorporated into TCA-precipitable protein relative to untreated incubations.

**Figure 3** Cell-binding ability of SWA11-SPDB-abrin A chain. SW2 cells exposed to different concentrations of SWA11-SPDB-abrin A chain (●) or unconjugated SWA11 (▲) were treated with flourescein-linked anti-mouse Ig and the cell-associated fluorescence was measured by flow cytometry.

**Figure 5** Cytotoxic effects of SWA11-SPDB-abrin A chain. SW2 cells in tissue culture were treated with SWA11-SPDB-abrin A chain (●), SWA11-SPDP-abrin A chain (○) abrin (■), and abrin A chain (□) at the concentrations shown. The effects on protein synthesis were judged from the amount of $^3$H-leucine incorporated by treated cells relative to that by parallel untreated cultures (>60,000 c.p.m.). Each point represents the mean of quadruplicate determinations. Error bars denote the standard deviations from the mean values except where smaller than the symbol used.

### *Cytotoxic effects of SWA11-SPDB-abrin A chain*

SWA11-SPDB-abrin A chain caused a concentration-dependent inhibition of $^3$H-leucine incorporation by SW2 cells in tissue culture. The results of a representative experiment are shown in Figure 5. The concentration at which $^3$H-leucine incorporation was inhibited by 50% ($IC_{50}$) was $1.2 \pm 0.6 \times 10^{-11}$ M (mean and standard deviation calculated from three separate experiments). At an IT concentration of $1 \times 10^{-9}$ M, $^3$H-leucine incorporation was decreased to less than 1% of that by untreated cells. The cytotoxic activity of the IT made with the hindred disulphide linker did not differ significantly from that of an analogous IT made with the standard unhindered linker, SWA11-SPDE-abrin A chain, which had an $IC_{50}$ of $0.8 \pm 0.6 \times 10^{-11}$ M. SWA11-SPDB-abrin A chain was approximately 220-fold less toxic than abrin, with an $IC_{50}$ of $5.4 \pm 1.7 \times 10^{-14}$ M and 160-fold more potent than unconjugated abrin A chain which had an $IC_{50}$ of $1.9 \pm 1.4 \times 10^{-9}$ M. Unconjugated SWA11 had no cytotoxic effects on SW2 cells at a concentration of $1 \times 10^{-7}$ M. In control experiments with the human T-lymphoblastoid cell line CEM, which does not express the antigen recognised by SWA11, the SWA11-SPDB-abrin A chain IT was no more toxic than unconjugated abrin A chain (not shown).

### *Toxicities of SWA11-SPDB-abrin A chain and abrin A chain*

The toxicity of SWA11-SPDB-abrin A chain to nude mice was determined by administering a single i.p. injection of sterile IT solution to mice at different doses. Mice treated with 15 μg of IT showed no evidence of ill effects or weight loss. At the 30 μg dose of IT, the mice showed a significant weight loss after 5 days with recovery after 9 days. An immediate and severe weight loss (>25% after 9 days) was observed in mice receiving 60 μg of IT; one mouse recovered and developed a peritoneal ascites. At the 120 μg dose level, both mice lost weight immediately and did not survive beyond 2 days after IT administration. The $LD_{50}$ was calculated to be 3.12 mg of IT per kg animal weight equivalent to 0.52 mg of conjugated A chain per kg. Similar toxic effects were observed with unconjugated abrin A chain. The $LD_{50}$ dose of abrin A chain was estimated to be 20.7 mg per kg.

### *Anti-tumour effects of SWA11-SPDB-abrin A chain*

Nude mice bearing established SW2 solid tumour xenografts were treated by a single i.v. injection of SWA11-SPDB-abrin A chain containing 9 μg of IT (50 pmoles) equivalent to approximately 15% of the $LD_{50}$ dose determined by i.p. administration of IT. Control groups of mice received equivalent doses of unconjugated abrin A chain (1.5 μg), of unconjugated SWA11 (7.5 μg), or of the carrier protein solution alone. Treatment of mice with these agents at the doses stated produced no weight losses or other signs of toxicity. The effects of the treatments on tumour growth were monitored by measuring the relative increase in tumour volume (Figure 6a). Tumour growth was not inhibited by abrin A chain alone compared to the treatment with carrier solution or untreated controls (omitted for clarity). The SWA11 Mab alone gave a slight tumour growth delay of 2 days although this was not significantly different from the growth of tumours in mice receiving abrin A chain. In contrast, tumour-bearing mice treated with SWA11-SPDB-abrin A chain demonstrated a growth delay of 7 days relative to those treated with abrin A chain only which was significant by Student's t-test ($P = 0.05-0.02$).

For the groups of tumour-bearing mice treated with the IT or the A chain, the mean tumour volumes at the time of injection were $114 \pm 37\ mm^3$ and $116 \pm 26\ mm^3$ respectively. Analysis of the data for the three mice in each of these two groups bearing the smallest tumours, $55 \pm 4\ mm^3$ and $54 \pm 9\ mm^3$ for the IT and A chain respectively, revealed a more pronounced anti-tumour effect (Figure 6b). Tumour-bearing mice treated with IT showed no increase in tumour size in the 7 days following IT injection and a significant ($P = 0.05-0.02$) tumour growth delay averaging 10 days.

In a further experiment, tumour-bearing mice were treated with a single i.v. injection of carrier-free abrin A chain at a dose of 3.1 mg $kg^{-1}$ equivalent to 15% of the $LD_{50}$ dose determined by i.p. administration of A chain. There was no significant difference in the growth rate of SW2 xenografts between groups of mice receiving the A chain or diluent alone.

**Figure 6** Anti-tumour effects of SWA11-SPDB-abrin A chain. **a**, Nude mice bearing established s.c. SW2 tumours received a single i.v. injection on day 0 of 9 μg SWA11-SPDB-abrin A chain (●), 7.5 μg SWA11 (▲) or 1.5 μg abrin A chain (□). Tumour diameters were measured at intervals following injection and the results were plotted as the percentage increase in tumour volume relative to the tumour volume at day 0. Error bars denote the standard deviations from the mean values. **b**, Experimental data for mice with tumours approximately 55 $mm^3$ in volume on day 0.

### Discussion

In this study, we have demonstrated the synthesis and purification of an IT designed for therapy of human SCLC which incorporates the aglycosyl abrin A chain linked via a hindered disulphide crosslinker SPDB to a mouse Mab SWA11 recognising an antigen highly associated with SCLC. The purified IT had a defined molecular composition corresponding to a 1:1 conjugate of SWA11 and abrin A chain. The chromatographic properties of the IT in solution were consistent with those of a monomeric globular protein of the expected molecular mass. SWA11-SPDB-abrin A chain fully retained the cell-binding capacity of the Mab and the ribosome-inactivating activity of the A chain. The IT exerted potent and selective toxic effects against the SW2 cell line in tissue culture and significantly delayed the growth of established SW2 tumour xenografts in nude mice treated with a single non-toxic dose.

The cytotoxic activity of SWA11-SPDB-abrin A chain was indistinguishable from that of an analogous IT made with the unhindered SPDP crosslinking reagent. This result was in accord with previous findings that increasing the resistance of the disulphide bond between Mab and A chain to chemical reduction had no deleterious effect upon IT activity (Worrell *et al.*, 1986; Thorpe *et al.*, 1988). The cytotoxic activity of the two SWA11-abrin A chain ITs was found to be identical to that of an analogous SWA11-SPDP-ricin A chain IT in parallel assays whereas abrin toxin and unconjugated abrin A chain were approximately 5-fold more toxic to the SW2 cell line than ricin and ricin A chain respectively (Derbyshire *et al.*, unpublished results). This suggests that the cytotoxic potency of the cell-binding ITs in this system was determined by the antigen-dependent route of entry into the target cell rather than by the inherent properties of the A chain.

The toxicity to nude mice of abrin A chain was increased by about 40-fold following conjugation to the SWA11 Mab via the SPDB crosslinker. The higher toxicity of the IT reflects, at least in part, the prolonged serum half-life of the abrin A chain coupled to Mab compared with the unconjugated A chain which is cleared rapidly from the bloodstream (Wawrzynczak *et al.*, 1990*a*). Model studies have shown that both the abrin A chain and the hindered disulphide crosslinker contribute to the stability of this type of IT to reduction by glutathione (Cumber *et al.*, unpublished results). The toxicity of abrin A chain ITs has not previously been reported but, in another study, the attachment of deglycosylated ricin A chain to a Mab via a different hindered crosslinker resulted in only a 7-fold increase in its toxicity to normal Balb/c mice (Thorpe *et al.*, 1988). This apparent difference in toxicity could have a number of causes: firstly, if the abrin A chain IT persisted in the bloodstream at a higher level leading to greater exposure of normal tissues; secondly, if the abrin A chain IT interacted preferentially with some essential normal tissue; thirdly, if strains of mice differed in their susceptibility to intoxication. These uncertainties can best be addressed in future by direct comparison of the pharmacokinetics, biodistribution and toxicities of abrin A chain and deglycosylated ricin A chain ITs made with the same Mab and crosslinker.

The anti-tumour efficacy of SWA11-SPDB-abrin A chain was tested in nude mice bearing established s.c. solid tumours derived from the SW2 human SCLC cell line. A single i.v. injection of the IT at a non-toxic dose induced a delay in tumour growth that could not be matched by unconjugated SWA11 or abrin A chain. The largest anti-tumour effects in the xenograft model were observed in the mice bearing the smallest tumours at the time of injection suggesting that such anti-SCLC ITs may be most effective against small tumour deposits and metastases present at an early stage in the disease or following reduction of tumour bulk by chemotherapy. The anti-tumour effects were modest given that the antigen recognised by SWA11 is highly expressed on the SW2 cell line (>99.9% cells positive by indirect immunofluorescence), that the pattern of antigen expression is highly similar between SW2 cells in tissue culture and in xenografts, and that SWA11-A chain ITs can selectively eliminate >99.9% of clonogenic SW2 cells in culture (Derbyshire *et al.*, unpublished results; Zangemeister-Wittke *et al.*, unpublished results). Further studies will aim to determine whether tumour progression resulted from the outgrowth of target antigen-negative or IT-resistant cells or from failure to eradicate target antigen-positive cells because of insufficient IT penetration, and to define the optimal dosing schedules for achieving maximal anti-tumour effects.

In summary, we have demonstrated that a selectively cytotoxic SWA11-abrin A chain IT made with a hindered disulphide crosslinker was capable of inducing a significant delay in the growth of a solid SCLC tumour xenograft in a nude mouse model at a single non-toxic dose. This is the first report describing the anti-tumour effects of an anti-SCLC A chain IT *in vivo*. The findings of this study have two major implications. Firstly, abrin A chain appears to be highly suitable for the production of therapeutic ITs. The cloning of the abrin gene and the demonstration of the expression of the A chain from *Escherichia coli* in catalytically active form (Wood *et al.*, 1991) will permit the further optimisation of the properties of abrin A chain ITs. Secondly, the antigen recognised by the SWA11 Mab is an effective target for therapy of SCLC with A chain ITs. This antigen has recently been cloned and characterised (Waibel *et al.*, unpublished results) enhancing the prospect of developing second generation anti-SCLC ITs with greater activity and selectivity.

We thank Drs C. Dean and L. Kelland for their critical appraisal of the manuscript. This work was supported by the Cancer Research Campaign, UK and the Swiss Cancer League FOR.802.87.1, FOR.302.89.2. The study forms part of the EEC Concerted Action on Drug Targeting: Immunoconjugates for Cancer Therapy.

### References

BLAKEY, D.C., WAWRZYNCZAK, E.J., STIRPE, F. & THORPE, P.E. (1987). Anti-tumour activity of a panel of anti-Thy1.1 immunotoxins made with different ribosome-inactivating proteins. In *Membrane-Mediated Cytotoxicity*, Bonavida, B. & Collier, R.J. (eds), pp. 195-202, A.R. Liss Inc: New York.

CUMBER, A.J., FORRESTER, J.A., FOXWELL, B.M.J., ROSS, W.C.J. & THORPE, P.E. (1985). Preparation of antibody-toxin conjugates. *Methods Enzymol.*, **112**, 207-225.

DERBYSHIRE, E.J. & WAWRZYNCZAK, E.J. (1991). Monoclonal antibodies recognising the cluster 2 antigen associated with human small cell lung cancer mediate the toxic effects of ricin A chain in an indirect assay of immunotoxin cytotoxicity. *Br. J. Cancer*, **63**, Suppl. XIV, 74-77.

FORRESTER, J.A., MCINTOSH, D.P., CUMBER, A.J., PARNELL, G.D. & ROSS, W.C.J. (1984). Delivery of ricin and abrin A chains to human carcinoma cells in culture following linkage to monoclonal antibody LICR-LOND-Fib75. *Cancer Drug Deliv.*, **1**, 283-292.

HWANG, K.M., FOON, K.A., CHEUNG, P.H., PEARSON, J.W. & OLDHAM, R.K. (1984). Selective antitumor effect on L10 hepatocarcinoma cells of a potent immunoconjugate composed of the A chain of abrin and a monoclonal antibody to a hepatoma-associated antigen. *Cancer Res.*, **44**, 4578-4586.

KNOWLES, P.P. & THORPE, P.E. (1987). Purification of immunotoxins containing ricin A chain and abrin A chain using Blue Sepharose CL-6B. *Anal. Biochem.*, **160**, 440-443.

OLSNES, S., REFSNES, K., CHRISTENSEN, T.B. & PIHL, A. (1975). Studies on the structure and properties of the lectins from *Abrus precatorius* and *Ricinus communis*. *Biochim. Biophys. Acta.*, **405**, 1-10.

SIVAM, G., PEARSON, J.W., BOHN, W., OLDHAM, R.K., SADOFF, J.C. & MORGAN, A.C. Jr (1987). Immunotoxins to human melanoma-associated antigen: comparison of gelonin with ricin and other A chain conjugates. *Cancer Res.*, **47**, 3169-3175.

SKILLETER, D.N., PRICE, R.J., PARNELL, G.D. & CUMBER, A.J. (1989). The low uptake of an abrin A chain immunotoxin by rat hepatic cells *in vivo* and *in vitro*. *Cancer Lett.*, **46**, 161-166.

SMITH, A., WAIBEL, R., WESTERA, G., MARTIN, A., ZIMMERMAN, A.T. & STAHEL, R.A. (1989). Immunolocalisation and imaging of small cell cancer xenografts by the IgG2a monoclonal antibody SWA11. *Br. J. Cancer*, **59**, 174-178.

SMITH, A., GROSCURTH, P., WAIBEL, R., WESTERA, G. & STAHEL, R.A. (1990). Imaging and therapy of small cell carcinoma xenografts using $^{131}$I-labelled monoclonal antibody SWA11. *Cancer Res.* (Suppl.) **50**, 980s-984s.

SMITH, A., WAIBEL, R. & STAHEL, R.A. (1991). Selective immunotherapy of small cell cancer xenografts using $^{131}$I-labelled SWA11 antibody. *Br. J. Cancer*, **64**, 263-266.

THORPE, P.E., BLAKEY, D.C., BROWN, A.N.F., KNOWLES, P.P., KNYBA, R.E., WALLACE, P.M., WATSON, G.J. & WAWRZYNCZAK, E.J. (1987). Comparison of two anti-Thy1.1 abrins A chain immunotoxins prepared with different cross-linking agents: anti-tumour effects, *in vivo* fate, and tumor cell mutants. *J. Natl. Cancer. Inst.*, **79**, 1101-1112.

THORPE, P.E., WALLACE, P.M., KNOWLES, P.P., RELF, M.G., BROWN, A.N.F., WATSON, G.J., BLAKEY, D.C. & NEWELL, D.R. (1988). Improved antitumor effects of immunotoxins prepared with deglycosylated ricin A chain and hindered disulfide linkages. *Cancer Res.*, **48**, 6396-6403.

WAWRZYNCZAK, E.J. (1991). Systemic immunotoxin therapy of cancer: advances and prospects. *Br. J. Cancer*, **64**, 624-630.

WAWRZYNCZAK, E.J., CUMBER, A.J., HENRY, R.V. & PARNELL, G.D. (1991*a*). Comparative biochemical, cytotoxic and pharmacokinetic properties of immunotoxins made with native ricin A chain, ricin $A_1$ chain and recombinant ricin A chain. *Int. J. Cancer*, **47**,130-135.

WAWRZYNCZAK, E.J., CUMBER, A.J., HENRY, R.V., MAY, J., NEWELL, D.R., PARNELL, G.D., WORRELL, N.R. & FORRESTER, J.A. (1990*a*). Pharmacokinetics in the rat of a panel of immunotoxins made with abrin A chain, ricin A chain, gelonin, and momordin. *Cancer Res.*, **50**, 7519-7526.

WAWRZYNCZAK, E.J., DERBYSHIRE, E.J., HENRY, R.V., PARNELL, G.D., SMITH, A., WAIBEL, R. & STAHEL, R.A. (1990*b*). Selective cytotoxic effects of a ricin A chain immunotoxin made with the monoclonal antibody SWA11 recognising a human small cell lung cancer antigen. *Br. J. Cancer*, **62**, 410-414.

WAWRZYNCZAK, E.J., DERBYSHIRE, E.J., HENRY, R.V., PARNELL, G.D., SMITH, A., WAIBEL, R. & STAHEL, R.A. (1991*b*). Cytotoxic activity of ricin A chain immunotoxins recognising cluster 1, w4 and 5A antigens associated with human small cell lung cancer. *Br. J. Cancer*, **63**, Suppl. XIV, 71-73.

WEIL, C.S. (1952). Tables for convenient calculation of median-effective dose ($LD_{50}$ or $ED_{50}$) and instructions in their use. *Biometrics*, **8**, 240–263.

WOOD, K.A., LORD, M.J., WAWRZYNCZAK, E.J. & PIATAK, M. (1991). Preproabrin: genomic cloning, characterization and the expression of the A chain in *Escherichia coli*. *Eur. J. Biochem.*, **198**, 723–732.

WORRELL, N.R., CUMBER, A.J., PARNELL, G.D., MIRZA, A., FORRESTER, J.A. & ROSS, W.C.J. (1986). Effect of linkage variation on pharmacokinetics of ricin A chain antibody conjugates in normal rats. *Anti-Cancer Drug Design*, **1**, 179–188.